# Draft Genome Sequence of Rhodococcus rhodochrous Strain G38GP, Isolated from the Madagascar Hissing Cockroach

**DOI:** 10.1128/MRA.00777-21

**Published:** 2021-10-07

**Authors:** Juan Guzman, Andreas Vilcinskas

**Affiliations:** a Department of Bioresources, Fraunhofer Institute for Molecular Biology and Applied Ecology, Giessen, Germany; b Institute for Insect Biotechnology, Justus-Liebig-University of Giessen, Germany; University of Maryland School of Medicine

## Abstract

Rhodococcus rhodochrous is a bacterial species with applications in biocatalysis and bioremediation. Here, we report the draft genome sequence of strain G38GP, isolated from the gut of the cockroach Gromphadorhina portentosa. The genome consists of 76 contigs, with a total length of 6,256,198 bp and a GC content of 67.82%.

## ANNOUNCEMENT

Fifty-two species of the genus *Rhodococcus* (*Nocardiaceae*: *Mycobacteriales*) with validly published names have been described (1). The type strain is Rhodococcus rhodochrous ATCC 13808^T^ (=DSM 43241^T^). *R. rhodochrous* strains are used as industrial biocatalysts (2–5). Here, we report the draft genome sequence of *R. rhodochrous* strain G38GP, isolated from the gut of the Madagascar hissing cockroach, Gromphadorhina portentosa Schaum.

Specimens of Gromphadorhina portentosa are maintained as model organisms for entomology courses at the Justus-Liebig-University of Giessen, Germany. The gut of a female cockroach was dissected, suspended in sterile 50% glycerol, and cut into small fragments using sterile surgical scissors. We then plated a 1:10 dilution onto solid medium comprising 0.2% chitosan, 0.1% l-arginine, 1.5% agar, 50 mg/liter nalidixic acid, and 100 mg/liter nystatin, supplemented with trace element and vitamin solutions as previously described (6). A single colony was recultured and the 16S rRNA gene amplified and sequenced as previously reported (6). The nearly complete 16S rRNA sequence of strain G38GP (1,366 bp; GenBank accession number MW167072) showed the highest similarity to *R. rhodochrous* NBRC 16069^T^ (99.85%; BBXP01000056), followed by Rhodococcus biphenylivorans TG9^T^ (99.34%; KJ546454). The strain G38GP was deposited in the DSMZ open strain collection (DSM 111905).

A single colony was cultured in tryptic soy broth for 72 h at 28°C, and DNA was extracted from the cell pellet using the Wizard genomic DNA purification kit (Promega, Madison, WI, USA). A DNA library was prepared using the Nextera XT library preparation kit (Illumina, San Diego, CA, USA), and 150-bp paired-end reads were generated on a HiSeq 4000 device by SNPsaurus (Eugene, OR, USA). The resulting 4,344,176 read pairs were submitted to GenBank under SRA accession number SRR15212339. Default parameters were used for all software unless otherwise specified. The reads were assembled *de novo* using MaSuRCA v3.4.2 without preassembly quality control, as recommended (7). The assembly was assessed using QUAST (8). The draft genome sequence of strain G38GP was submitted to GenBank under accession number JADKNM000000000 (BioSample accession number SAMN16606023).

**FIG 1:**
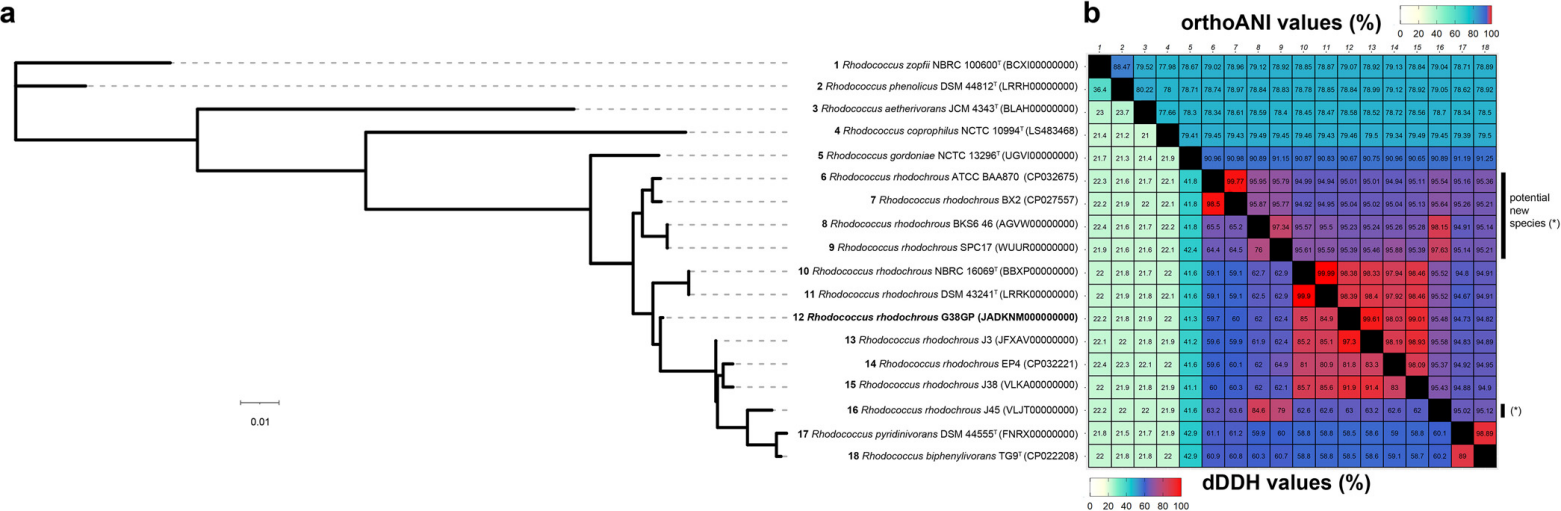
Genome-based taxonomy of the strain *R. rhodochrous* G38GP. (a) Phylogenomic tree showing the position of strain G38GP and 17 related *Rhodococcus* strains. (b) Overall genome relatedness metrics (orthologous ANI [orthoANI] and dDDH values) showing strain G38GP to be a member of the species *R. rhodochrous*. The phylogenomic tree was constructed using bcgTree (14) and was inferred using IQ-TREE v2.0.3 (15), with automatically optimized models for each partition and bootstraps calculated over 10^5^ generations. The dDDH and ANI values were calculated using the Genome-to-Genome Distance Calculator (12) and the OrthoANI Java tool (13).

Genome annotation was performed using the NCBI Prokaryotic Genome Annotation Pipeline (9). The draft genome sequence of *R. rhodochrous* G38GP consists of 76 contigs, with a total length of 6,256,198 bp and an *N*_50_ value of 292,056 bp. The GC content is 67.82%. Genome annotation revealed a total of 5,797 genes, including 5,471 protein-coding sequences, 69 tRNA genes, and 14 rRNA genes. The genome sequence was found to be 98.9% complete using BUSCO v4.1.2 (10), based on the actinobacteria_phylum_odb10 lineage data set. Genome analysis at TYGS (11) confirmed that isolate G38GP belongs to the species *R. rhodochrous*. A phylogenomic tree showed that G38GP is closely related to the type strain *R. rhodochrous* NBRC 16069^T^ (=DSM 43241^T^ = NCTC 10210^T^), according to the branch length calculations (Fig. 1a). Digital DNA-DNA hybridization (dDDH) and average nucleotide identity (ANI) values confirmed the taxonomic position of G38GP within the species *R. rhodochrous*, with values of ∼85% and ∼98% relative to the type strain, higher than the thresholds of 70% and 95 to 96% for species delimitation (12, 13) (Fig. 1b).

### Data availability.

The GenBank/EMBL/DDBJ accession numbers of the 16S rRNA gene and draft genome sequences of strain Rhodococcus rhodochrous G38GP (=DSM 111905) are MW167072.1 and JADKNM000000000.1, respectively. The raw sequencing data were deposited under SRA accession number SRR15212339.
